# The role of probiotics in modulation of the gut-brain axis: a prospective therapy for depression and mood disorders

**DOI:** 10.3389/fphar.2025.1709060

**Published:** 2026-01-26

**Authors:** Hui Wang, Yining Chen, Allen Zhao, Zhongxia Shen, Yu Zhang

**Affiliations:** 1 Department of Pharmacology, School of Pharmacy, Nantong University, Nantong, China; 2 Department of Neuroscience and Cell Biology, Robert Wood Johnson Medical School, Rutgers Biomedical and Health Sciences, Piscataway, NJ, United States; 3 The Experimental High School Attached to Beijing Normal University, Beijing, China; 4 Department of Biochemistry, College of Agricultural and Life Sciences, University of Wisconsin-Madison, Madison, WI, United States; 5 Department of Sleep Medical Center, Huzhou Third Municipal Hospital, the Affiliated Hospital of Huzhou University, Huzhou, China; 6 Department of Gastroenterology, Henan Provincial People’s Hospital, People’s Hospital of Zhengzhou University, Zhengzhou, China

**Keywords:** gut-brain axis, hypothalamic-pituitary-adrenal axis, major depressive disorder, microbiota, probiotics

## Abstract

The gut-brain axis is a bidirectional pathway linking the gastrointestinal microbiota to neurological functions. While its significance in the pathogenesis of gastrointestinal conditions is well-documented, emerging evidence indicates that dysbiosis of the gut microbiota could also be implicated in various neuropsychiatric disorders, specifically major depressive disorder (MDD). MDD represents a debilitating illness that accounts for a significant portion of global disability. Although numerous medications have been developed to manage depression, they are frequently plagued by variable efficacy and unpleasant adverse effects. The inconsistency of antidepressant effects highlights the complexity and poorly understood pathophysiology underlying this condition. Recent studies suggest that MDD may involve disruptions in the gut-brain axis via gut dysbiosis, induction of inflammation, metabolic disturbances of neuroactive substances, and dysregulation of the hypothalamic-pituitary-adrenal axis, along with the autonomic and enteric nervous systems. Given the direct and indirect connections between the microbiota and these physiological processes, probiotics are increasingly being explored as a prospective therapeutic option for MDD. Multiple probiotic formulations have shown promise in both preclinical and clinical settings, demonstrating effectiveness in attenuating symptoms associated with MDD. This review provides an overview of the pathophysiologic attributes of MDD, with particular focus on disturbances along the gut-brain axis, and investigates current findings regarding the role of probiotics in addressing these challenges. We conclude by identifying persistent gaps in the literature and proposing directions for future studies.

## 1 Introduction

**FIGURE 1:**
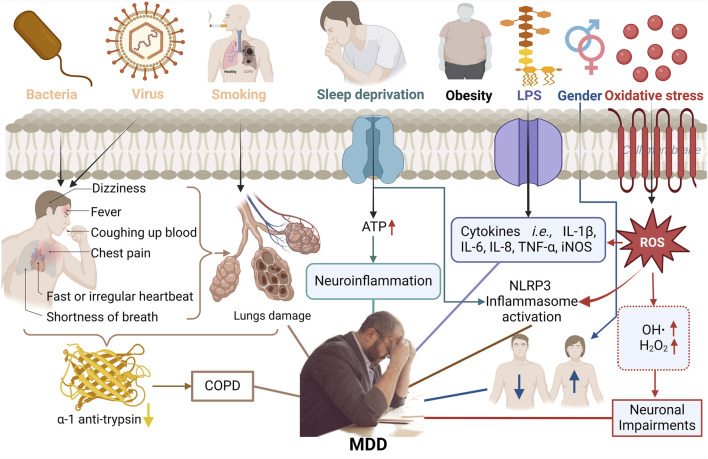
Risk factors for MDD include individual conditions such as smoking, COPD, sleep deprivation, obesity, environmental milieu, gender, oxidative stress, and genetic susceptibility.

Major depressive disorder (MDD) constitutes a leading cause of disability and suicide, characterized by enduring negative thoughts and emotions, which ranks among the most burdensome psychiatric diseases worldwide (Ménard et al., 2016; Malhi and Mann, 2018). Statistically, the overall incidence of MDD is approximately 6%, with one in five people experiencing a depressive episode at some point during their lifetime. While the prevalence is comparable across both developed and developing countries, women are twice as likely as men to be at risk (Malhi and Mann, 2018). The risk factors for MDD are multifaceted and encompass individual conditions such as gender (Salk et al., 2017), smoking (Mathew et al., 2017), obesity (Milaneschi et al., 2019), chronic obstructive pulmonary disease (COPD) (Prasad, 2020), sleep deprivation (Mitter et al., 2022), unhealthy diet (Quirk et al., 2013), environmental milieu, and genetic susceptibility (Figure 1) (Penner-Goeke and Binder, 2019). Most of these factors can trigger chronic inflammation and stress responses within the body, particularly resulting in elevated levels of proinflammatory cytokines (e.g., TNF-α, IL-1β, and IL-6) as well as corticosterone, which can further exacerbate depression and vice versa (de Kloet et al., 2016; Weina et al., 2018; Qi et al., 2024).

Mounting studies have shown that MDD is an inflammatory disease, with gut microbiota playing a critical role in systemic inflammation and the pathogenesis of MDD (Kohler et al., 2016; Zhang et al., 2020). One perspective is that gut dysbiosis can increase the permeability of the intestinal membrane, allowing harmful substances, such as lipopolysaccharide (LPS), to enter the bloodstream and activate the immune system and the hypothalamic-pituitary-adrenal (HPA) axis through various inflammatory mediators (Naseribafrouei et al., 2014; Foster and McVey Neufeld, 2013; Dinan et al., 2017; Dinan et al., 2013). Clinical antidepressants remain the first-line treatments that target the metabolism of monoamine neurotransmitters, including selective serotonin reuptake inhibitors (SSRIs), serotonin-norepinephrine reuptake inhibitors (SNRIs), and norepinephrine-dopamine reuptake inhibitors (NDRIs) (Chang et al., 2022). However, gut dysfunction is among the noted side effects of these psychotropic drugs, primarily attributed to the diminished activity of serotonin-selective reuptake transporters. In addition, approximately 50% of patients do not achieve adequate treatment outcomes with available therapies because of the heterogeneity of MDD. These challenges underscore the urgent necessity for the development of innovative therapeutic strategies (Wang et al., 2021).

Probiotics are defined as live microorganisms that, when administered in adequate amounts, provide health benefits on the host (Hill et al., 2014). While probiotics are widely employed in clinical practices as adjuvant therapies for gastrointestinal conditions such as constipation (Yoon et al., 2018), antibiotic-associated diarrhea (Esposito et al., 2018), and irritable bowel syndrome (Oh et al., 2019), recent findings also suggested their translational potential in neuropsychiatric disorders, functioning via the gut-brain axis (Huang et al., 2016). From this, the emergence of certain probiotics, referred to as “psychobiotics,” could typically confer mental benefits, promising therapeutic options for dreary mental health challenges (Dinan et al., 2013; Smith et al., 2021; Dinan and Cryan, 2017). Notably, recent studies demonstrated that human-origin probiotic cocktails could enhance cognitive performance and reduce neuropathological features in an APP/PS1 mouse model of Alzheimer’s disease (Prajapati et al., 2025a). Specifically, the human-origin probiotics exert their effects by modulating gut microbial composition, enhancing short-chain fatty acids (SCFAs) metabolism, and restoring the integrity of both the intestinal and blood–brain barriers (BBB), thereby attenuating microglial activation and neuroinflammation (Prajapati et al., 2025a; Prajapati et al., 2023). These findings elucidate a shared mechanistic framework underlying probiotic therapeutic strategies targeting the gut microbiome, highlighting a broader clinical potential of probiotics in the management of MDD.

This review provides a comprehensive overview of the physiological and pathological alterations in MDD, particularly underpinning the compelling linkage of MDD to the disordered brain-gut-microbiome axis. It synthesizes well-established evidence concerning the intricate interplays across microbial, immune, metabolic, endocrine, and neural connections. Further, we concentrate exclusively on preclinical and clinical studies that involve single-strain probiotic interventions, primarily featuring species of *Bifidobacterium* and *Lactobacillus*, and reinforce the integration of strain-specific mechanisms of action with critical study parameters, such as dosage, duration, and pathophysiology outcomes. In addition, we emphasize next-generation probiotics (e.g., Faecalibacterium prausnitzii, Akkermansia muciniphila, and Clostridium butyricum) for the emerging evidence of their antidepressant promise. Moreover, Complementary microbiome-based therapies alongside probiotic interventions are also explored, including prebiotics, synbiotics, postbiotics, and fecal microbiota transplantation (FMT) within the context of MDD. The studies included in this review are selected based on specific criteria: (1) preclinical investigations using animal models of stress and clinical trials involving human participants assessing depressive symptoms; (2) publications from the past decade to capture the latest advancements in gut-brain axis research in relation to neuropsychiatric disorders; (3) studies focusing on specific probiotic strains or microbiome-based therapies that demonstrate significant effectiveness in addressing depression; and (4) peer-reviewed publications to ensure scientific rigor. Studies were excluded if they lacked direct relevance to MDD or if they focused solely on gastrointestinal outcomes without considering their neuropsychiatric implications.

## 2 Altered brain-gut-microbiome axis in MDD

**FIGURE 2:**
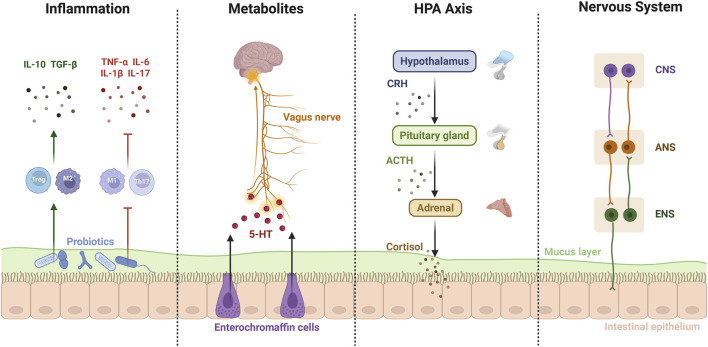
The bidirectional gut-brain communication depends on a multifaceted interplay of immune, metabolic, endocrine, and neural pathways.

The human gastrointestinal tract accommodates approximately 3.8 × 10^13^ bacterial cells with high abundance and diversity, in which the composition of gut microbiota changes dynamically throughout different periods of life (Sun et al., 2020). Disorders in the gut microbiome, particularly in terms of microbial diversity and taxa, can significantly influence brain pathogenesis and vice versa (Zhu et al., 2020; Mohajeri et al., 2018; Rinninella et al., 2019a). This brain-gut-microbiome axis has long been appreciated for its profound association with various neuropsychiatric diseases, including MDD, Parkinson’s disease, Alzheimer’s disease, autism, anxiety, and schizophrenia (Cryan et al., 2019; Yang X. et al., 2024). Current research delineates at least four interdependent ways for bidirectional gut-brain communication (Figure 2): 1. immune pathway, via inflammatory cytokines; 2. metabolic pathway, including substances such as SCFAs and tryptophan (TRP) associated with commensal bacteria; 3. endocrine pathway, primarily through hormones implicating the HPA axis; 4. neural connection, mainly involving the enteric nervous system (ENS) and autonomic nervous system (ANS) (Farzi et al., 2018; Belkaid and Hand, 2014; Carabotti et al., 2015). The pathogenesis of MDD is complicated and not yet well-illuminated, which contributes to the ongoing lack of effective therapies (Ménard et al., 2016). The dysfunctional brain-gut-microbiome axis has thus garnered significant attention in the context of MDD, where dysbiosis of gut microbiota, dysregulation of the immune system, disruption of neuroactive substance metabolism, and disorders of the HPA axis, ANS, as well as ENS, are all intricately woven together (Wohleb et al., 2016; Otte et al., 2016; Zheng et al., 2016).

### 2.1 Dysbiosis of gut microbiota

In healthy individuals, the gut microbiota is primarily composed of two dominant phyla representing 90% of the microbial community, *Firmicutes* (e.g., *Clostridium* and *Lactobacillus*) and *Bacteroidetes* (e.g., *Bacteroides* and *Prevotella*), with less abundant phyla such as *Actinobacteria* (e.g., *Bifidobacterium*) and *Proteobacteria* (Rinninella et al., 2019a). These microorganisms are indispensable for producing beneficial metabolites, such as SCFAs, vitamins, and neuroactive substances (like serotonin), which are critical for maintaining intestinal barrier integrity, immune system function, and mood state (Haase et al., 2018; Winter et al., 2018; Rinninella et al., 2019b; Rowland et al., 2018; Peng et al., 2009). Recent findings have revealed that MDD typically exhibits decreased diversity and altered taxonomic composition of the gut microbiome, as confirmed by large-scale metagenomic analyses (Hu et al., 2023). A multi-cohort meta-analysis involving 1,054 participants across Europe reported that MDD subjects showed an average reduction in the Shannon diversity index and significant depletion of commensal genera, including *Faecalibacterium*, *Coprococcus*, *Dialister*, and *Bifidobacterium*. These taxa were tightly associated with enhanced microbial capacity for butyrate synthesis and positively correlated with quality-of-life measures (Valles-Colomer et al., 2019). Further corroborating these findings, a study (n = 261) found that MDD patients had decrease in observed species richness and Chao1 diversity, respectively. This study further noted a shift toward proinflammatory microbial profiles, characterized by increased populations of *Eggerthella*, *Alistipes*, and *Flavonifractor*, which are linked to reduced SCFA production and downregulation of microbial genes related to tryptophan (TRP) and gamma-aminobutyric acid (GABA) biosynthesis (Zhong et al., 2022).

Dysbiosis of the gut microbiota is proposed to be linked to MDD in several mechanisms. Firstly, it can result in lower production of SCFAs in the intestine, which could promote gut permeability by regulating the tight junction proteins between intestinal epithelial cells (IECs). The compromised intestinal barrier further allows endotoxins (e.g., LPS) to access the bloodstream and trigger chronic inflammation, a defining characteristic of MDD (Kohler et al., 2016; Peng et al., 2009; Raison et al., 2006; Felger et al., 2013a; Berk et al., 2013). Secondly, gut dysbiosis leads to a reduction of commensal microorganisms and an overgrowth of gut pathogens that express pathogen-associated molecular patterns (PAMPs). These PAMPs, recognized by pattern-recognition receptors such as the toll-like receptors (TLRs) family expressed on immune cells, could activate intestinal immune responses, elevate proinflammatory cytokine production, and in turn, exacerbate microbiota dysbiosis (Wang et al., 2019; Perez-Lopez et al., 2016; Wang X. et al., 2022). Moreover, altered gut microbiota could directly influence mental state due to abnormal metabolism of neuroactive substances, like monoamine neurotransmitters. It is evident that gut microbial transplantation from MDD patients to germ-free rats could induce depressive-like symptoms, and that effective probiotics could exhibit antidepressant-like effects to rescue gut dysbiosis in MDD animals and human trials (Wei et al., 2019; Tian et al., 2020; Kelly et al., 2016). Together, prevailing research underscores the significant role of gut dysbiosis in the pathophysiology of MDD, while acknowledging limitations, including variability in microbial profiles across different populations and a lack of longitudinal studies.

### 2.2 Dysregulation of the immune system

The intestinal immune system is considered a vital component of the integrated intestinal barrier function, interacting with the gut microbiota to maintain homeostasis via sophisticated mechanisms (Qiao et al., 2024). In a healthy intestinal ecosystem, commensal gut microbiota play a crucial role in preventing the colonization and overgrowth of pathogens by competing for limited space and nutrients within the lumen, as well as stimulating the secretion of antimicrobial factors from Paneth cells (Vaishnava et al., 2008). Besides this, the commensal *Bacteroides fragilis,* with its capsular polysaccharide A (PSA), can interact with TLR-2 to induce the migration of regulatory T cells, which could prompt mesenteric lymph nodes to release anti-inflammatory cytokines into the gut (Perez-Lopez et al., 2016). Similarly, commensal *Bacteroides thetaiotaomicron* can also alleviate gut inflammation by selectively counteracting proinflammatory factors like NF-κB (Jia et al., 2021). In addition, mucosa-specific antibody IgA in the gut can further preclude immune reactions from becoming hyperactive and demonstrate anti-inflammatory effects (Peterson et al., 2007). Collectively, a balanced composition of gut microbiota helps leverage immune responses to a normal extent and, in turn, contributes to a healthy population of commensal microorganisms.

In the context of MDD, the abundance and diversity of commensal microorganisms in the gut dramatically decline. This reduction can lead to the overgrowth of harmful pathogens and diminish the inhibitory effect of gut microbiota on immune responses. Consequently, these alterations could further contribute to a proinflammatory state in the host, characterized by elevated levels of proinflammatory cytokines in the bloodstream. A similar pattern is observed in irritable bowel syndrome (IBS), which is also marked by decreased abundance and diversity of gut microbiota, along with robust mucosal inflammation (Bhattarai et al., 2017; Ng et al., 2018). These parallels may explain the frequent manifestation of depressive symptoms in IBS patients and the noted efficacy of antidepressants in treating IBS in clinical practice. Augmented levels of proinflammatory cytokines have been detected and verified in both MDD animal models and patients, which have been regarded as serum hallmarks of the disorder. Dowlati et al. (2010) performed a meta-analysis of 24 trial studies to identify that TNF-α and IL-6 are the most elevated proinflammatory cytokines in MDD patients. Additionally, Zhao et al. (2019) demonstrated that LPS-stressed mouse models of depression exhibited increased levels of TNF-α, IL-1β, and IL-6 in both serum and brain tissues.

Notably, the immune system plays a significant role in stress-related psychopathologies via the intricate microbiome-neuronal-immune axis (Zhao et al., 2025; Bastiaanssen et al., 2020). This complex pathway encompasses the interplay among the immune system, gut microbiota, and the nervous system, which is increasingly highlighted in MDD. Dysbiosis of the gut microbiota alters microbial-derived metabolites (such as SCFAs, neurotransmitters, and LPS), leading to increased intestinal permeability and translocation of microbial-associated molecular patterns (MAMPs), which subsequently trigger peripheral immune activation (e.g., elevated IL-1β, IL-6, TNF-α) and microglial priming in the central nervous system (Wang I-C. et al., 2024). These immune signals interact with neuronal circuits through several routes: (i) they dysregulate the neuroendocrine system via the hypothalamic-pituitary-adrenal (HPA) axis, which further modulates neurotransmitter signaling and neural plasticity (Li et al., 2025); (ii) they stimulate afferent fibers of the vagus nerve with gut-derived neuroactive metabolites, thereby impacting brain regions associated with mood regulation (Foster et al., 2021); and (iii) they enable direct immune-to-brain communication as cytokines can cross a compromised blood-brain barrier (BBB) to prompt microglial activation. Within the overall framework of MDD, an imbalanced gut microbiota can trigger peripheral immune signaling and systemic inflammation, thereby further increasing BBB permeability and neuroinflammation in the CNS. In this state, microglia transition from a homeostatic to a pro-inflammatory M1 phenotype, releasing cytokines and reactive oxygen species (ROS). Such neuroinflammation, along with neurotransmitter depletion, consequently impairs brain-derived neurotrophic factor (BDNF) expression, synaptic plasticity, neurogenesis, and neuronal survival, ultimately manifesting as the behavioral and cognitive symptoms of depression (Mehta et al., 2025). The integration of microbial, immune, and neural signaling within the microbiome-neuronal-immune axis could also provide a comprehensive mechanistic platform for understanding how probiotic interventions could potentially restore depression-related pathways by modulating the gut microbiota.

Echoing this integrated mechanistic platform, recent evidence supports that administration of probiotics could effectively aid in the management of MDD (Tian et al., 2020; Kelly et al., 2016; Vaishnava et al., 2008; Peterson et al., 2007; Bhattarai et al., 2017; Ng et al., 2018; Dowlati et al., 2010; Zhao et al., 2019; Liu et al., 2016). Advances in multi-omics research have substantially enhanced the understanding of how probiotics could generate psychobiotic effects by modulating neurotransmission, metabolic pathways, and immune signaling in mood disorders. Metagenomic analyses revealed that probiotic administration enriches microbial genes involved in the biosynthesis and metabolism of critical neuroactive compounds, including tryptophan, glutamate, and dopamine-related intermediates (Bosi et al., 2020; Sun et al., 2022). These microbial changes are mirrored in metabolomic profiles, which consistently demonstrate elevated levels of serotonin precursors (e.g., 5-hydroxytryptophan), dopamine metabolites, and GABA in fecal or plasma samples (Yong et al., 2020; Teng et al., 2021). Concurrent transcriptomic analyses of host tissues further indicate upregulation of key enzymes like TPH1 and glutamate decarboxylase (GAD), suggesting enhanced serotonergic and GABAergic tones (Miri et al., 2023). Moreover, probiotics have also been shown to promote the abundance of SCFAs-producing taxa, such as Bifidobacterium, Faecalibacterium, and the Lachnospiraceae group. These SCFAs could activate signaling cascades such as GPR41/43 and inhibit histone deacetylases, which contribute to improved neuroplasticity, homeostasis of the HPA-axis, and anti-inflammatory responses (Sun et al., 2016; Li et al., 2022; Qiao et al., 2025). Simultaneously, integrated multi-omics datasets highlight that probiotics could effectively suppress inflammation-associated immune responses. Metabolomic profiles uncovered reduced levels of endotoxins and proinflammatory lipids, while host transcriptomics revealed consistent downregulations of TLR4/NF-κB signaling and proinflammatory cytokines such as IL-6, TNF-α, and CRP (Li et al., 2022; Bhatia et al., 2022; Xue et al., 2017). Taken together, these coordinated changes in microbial, metabolic, and transcriptional profiles reinforce the understanding that immune dysregulation is a pervasive phenomenon in the context of MDD pathophysiology, further highlighting the promising role of probiotics within the intricate interactions of the microbiome-neuronal-immune axis.

### 2.3 Disturbance of neuroactive substances

The disturbance in the metabolism of neuroactive substances is a fundamental characteristic of MDD, particularly concerning SCFAs, brain-derived neurotrophic factor (BDNF), TRP, dopamine (DA), glutamate (Glu), and gamma-aminobutyric acid (GABA) (Wang L. et al., 2022).

SCFAs, such as butyrate, propionate, and acetate, are metabolites generated through the fermentation of indigestible macronutrients, primarily dietary fibers, by specific commensal microbiota in the gut (Xiao et al., 2021). One important species involved is *Akkermansia muciniphila*, known for breaking down mucin within the intestinal lining and producing propionate as a metabolic byproduct (Sudhakaran et al., 2022). Other key species, mainly *Faecalibacterium prausnitzii, Eubacterium rectale, Eubacterium hallii,* and *Ruminococcus bromii,* are the most significant contributors to butyrate production in the intestine (Louis et al., 2010). SCFAs provide numerous physiological benefits in the gut, among which butyrate is particularly noteworthy for enhancing intestinal barrier integrity and sustaining gut homeostasis by upregulating tight junction expressions like claudin-1 and zonula occludens-1 (Wang et al., 2012). Beyond their roles in gut health, SCFAs have been documented to modulate the mucosal immune system, cross the blood-brain barrier (BBB), and deliver neuroprotective and antidepressant effects (Perry et al., 2016; Han et al., 2014). For instance, SCFAs can influence the sympathetic nervous system by acting on G protein-coupled receptor 41 (GPR41), which plays a pivotal role in regulating metabolic homeostasis (Kimura et al., 2011). They can also directly attenuate immune responses induced by LPS, which could activate inflammatory signaling through TLR-4 on immune cells, leading to the activation of NF-κB and subsequent proinflammatory cytokine release. Specifically, butyrate has been shown to mitigate this inflammatory cascade by decreasing LPS translocation, thus lessening systemic inflammatory responses (Manco et al., 2010; Liu et al., 2014; Nastasi et al., 2015). It is noted that individuals with MDD typically exhibit an imbalanced gut microbiota composition with lower levels of intestinal SCFAs, potentially associated with the pathophysiology of the disorder. A trial study by Skonieczna-Żydecka et al. (2018) revealed that fecal SCFA levels in MDD patients were significantly reduced compared to healthy controls, particularly regarding acetic and propionic acids. Further, SCFA levels demonstrated a negative correlation with the severity of depressive symptoms per Beck’s Depression Inventory scores. Prior research suggested that the administration of butyrate in mice yields significant antidepressant effects through the upregulation of BDNF expression (Schroeder et al., 2007). Notably, Bambury et al. (2018) emphasized that promoting SCFA production should be a critical criterion in identifying and selecting effective psychobiotic strains for patients with psychiatric disorders. This perspective underscores the therapeutic significance of SCFAs in modulating mental health and broader systemic effects.

BDNF is a key regulatory protein involved in neurogenesis and neuroplasticity (Wu ZH. et al., 2022). Dysregulation of BDNF has been linked to MDD, as evidenced by both animal and postmortem studies (Malhi and Mann, 2018; Zhou et al., 2022). Notably, the role of BDNF in MDD may vary across distinct cerebral regions (Zhang et al., 2016). The deficit expression of BDNF in the forebrain or hippocampus can significantly impair the efficacy of antidepressants, especially in animal behavioral studies (Björkholm and Monteggia, 2016; Chen et al., 2023). Further, BDNF infusions into the midbrain or hippocampus have shown considerable behavioral improvements in rat depression models (Zhong et al., 2019). Conversely, elevated BDNF levels in the ventral tegmental area (VTA) and nucleus accumbens (NAc) have been associated with the development of MDD (Eisch et al., 2003); the selective removal of BDNF in the VTA-NAc region appears to produce antidepressant-like effects comparable to those of traditional antidepressants (Berton et al., 2006). These findings in the VTA-NAc regions contrast with the antidepressant benefits of BDNF observed in the forebrain and the hippocampus. Mechanistically, the chronic inflammatory state associated with MDD could account for decreased BDNF levels in the hippocampus. The peripheral immune system communicates with the brain through proinflammatory cytokines, which are found to be elevated in the bloodstream of MDD patients and animal models. In response, immune microglia in the brain release additional proinflammatory cytokines, exacerbating inflammation in specific cerebral regions like the hippocampus (Patterson, 2015). *In vivo* findings have confirmed that proinflammatory states can cause a decline in BDNF expression in the hippocampus at both the mRNA and protein levels (Zhang et al., 2014). Moreover, declined plasma levels of BDNF have also been noted to correlate with the severity of depressive symptoms. A study by Lee et al. (2007) discovered that MDD patients exhibit significantly lower plasma BDNF levels compared to healthy controls, particularly among those experiencing recurrent episodes. Notably, Kim et al. (2007) suggested a connection between low plasma BDNF levels and suicidal behavior, further implying the potential of BDNF as a critical biomarker for depressive disorders.

TRP, a precursor to serotonin (5-HT), is another crucial substance disrupted in MDD that usually exhibits reduced 5-HT activity in the brain (Yin et al., 2024). As one of the nine essential dietary amino acids for mammals, TRP plays a critical role in maintaining gut microbiota balance and immune homeostasis (Dodd et al., 2017). The body metabolizes TRP primarily through the kynurenine (KYN) pathway, which generates KYN and its derivatives—such as kynurenic acid (KYNA) and quinolinic acid (QA)—as well as through the serotonin pathway, leading to the production of serotonin and melatonin, critical neurotransmitters regulating mood, sleep, appetite, and pain (Tu et al., 2021). It is important to note that a small fraction of free plasma TRP (10%–15% of the total, unbound to albumin) can cross the blood-brain barrier (BBB) and be converted to 5-HT in the brain (Comai et al., 2020; Wang Y. et al., 2022). From this, it is reasonable to hypothesize that plasma TRP levels are lower in MDD patients compared to healthy controls. In line with this hypothesis, a meta-analysis by Ogawa et al. (2014), which included 24 studies, found significantly lower plasma TRP levels in MDD patients than in healthy controls, with particularly marked reductions in untreated cases. Several factors influence TRP concentrations in the bloodstream, including the activities of tryptophan 2,3-dioxygenase (TDO) and indoleamine 2,3-dioxygenase (IDO). Both enzymes catalyze the initial step of the KYN pathway, accounting for approximately 95% of TRP catabolism (Comai et al., 2020). IDO activity is elevated in proinflammatory conditions, such as those characterized by high levels of interferon-γ and TNF-α, which are commonly observed in MDD. Increased IDO activity could lead to heightened TRP degradation via the KYN pathway, subsequently reducing plasma TRP levels (Zhang et al., 2016; Chen et al., 2022; Schröcksnadel et al., 2006). Furthermore, the elevated TRP catabolism could result in an imbalance between QA and KYNA, a significant factor contributing to the pathophysiology of MDD. QA, an agonist of N-methyl-D-aspartate (NMDA) receptors, can induce excitotoxic neuronal damage, whereas KYNA serves as a neuroprotective NMDA receptor antagonist (Formolo et al., 2023; Myint et al., 2007). In the context of MDD, the increased degradation of TRP via the KYN pathway, driven by proinflammatory cytokines, likely surpasses the conversion of TRP to 5-HT in the brain and could concurrently increase neurotoxic QA activities and/or decrease neuroprotective KYNA production. Additionally, specific gut bacterial strains are also vital to modulating host TRP metabolism, referred to as the microbial metabolic pathway. This pathway can produce several TRP metabolites in the gut, such as 5-HT, indole, and its derivatives, which impact multiple host physiological processes. Notably, the majority of 5-HT in the body is synthesized by enterochromaffin cells in the gastrointestinal (GI) tract, which regulates gut mobility and secretion (Comai et al., 2020; Clarke et al., 2013). Indole and its derivatives act as ligands for the aryl hydrocarbon receptor (AhR) or the pregnane X receptor (PXR), exhibiting antibacterial and anti-inflammatory effects as well as maintaining gut barrier integrity and epithelial cell function (Roager and Licht, 2018). Moreover, gut bacteria such as *Lactobacillus johnsonii* and *Bifidobacterium infantis* could also modulate the activities of QA and KYNA (Clarke et al., 2013; Reigstad et al., 2015; Wikoff et al., 2009; Yano et al., 2015; Kennedy et al., 2017). These properties can collectively influence the pathophysiology of MDD via the brain-gut-microbiome axis. Intriguingly, certain effective probiotics have been demonstrated to promote circulating TRP levels and inhibit TRP catabolism through the KYN pathway, further reinforcing the significance of disturbed TRP metabolism in MDD (Rudzki et al., 2019).

Anhedonia, a hallmark feature of MDD, refers to the inability to experience pleasure, which is closely linked to the mesolimbic dopamine (MDA) system, particularly in the VTA-NAc region. Neurotransmitter dopamine (DA) and its related reward circuit play a key role in modulating pleasurable experiences, directly impacting the symptomatology of anhedonia (Belujon and Grace, 2017; Der-Avakian and Markou, 2012; Nestler and Carlezon, 2006). It is evident that the MDA system is dysfunctional with a downregulation of the DA reward circuit in MDD patients and animal models (Yadid and Friedman, 2008). For instance, Pruessner et al. (2004) indicated that depressive patients exhibit relatively insufficient dopaminergic activity in the ventral striatum, which encompasses the NAc. Further investigations utilizing learned helplessness rat models demonstrated lower densities of DA-1 and DA-2 receptors within the NAc (Yaroslavsky et al., 2006). Interestingly, chronic inflammatory conditions can also impede dopaminergic neurotransmission via multiple mechanisms, including impaired dopamine synthesis, packaging, and release (Nutt, 2006). For instance, the peripheral administration of proinflammatory cytokine IFN-γ in primates could decrease dopamine release in the striatum (Felger et al., 2013b). All these findings align with the significant challenges in treating anhedonia with SSRIs and lend support to the clinical practices of NDRIs in managing MDD.

In addition to monoaminergic dysfunction, disturbances in the Glu and GABA systems have also been extensively studied in relation to MDD. Glu, synthesized in neurons from intermediates of the tricarboxylic acid cycle, can bind to receptors such as NMDA to exert excitatory effects. As the primary precursor, Glu can be further converted into GABA in inhibitory GABAergic neurons that express the enzyme GAD (Lener et al., 2017). Utilizing proton magnetic resonance spectroscopy (MRS), clinical studies have shown reduced levels of Glu and GABA in specific cortical regions, such as the prefrontal cortex and anterior cingulate cortex, among MDD patients (Hasler et al., 2007; Price et al., 2009). These findings are corroborated by evidence suggesting that certain antidepressants could antagonize NMDA glu receptors and increase cortical GABA levels (Li, 2020). Interestingly, the potential of GABA-producing probiotics to deliver antidepressant effects in mice, comparable to the first-line SSRI antidepressant fluoxetine, further appreciates the brain-gut-microbiome axis in developing effective treatments for depression (Yunes et al., 2020).

### 2.4 Disorder of the hypothalamic-pituitary-adrenal axis

**FIGURE 3:**
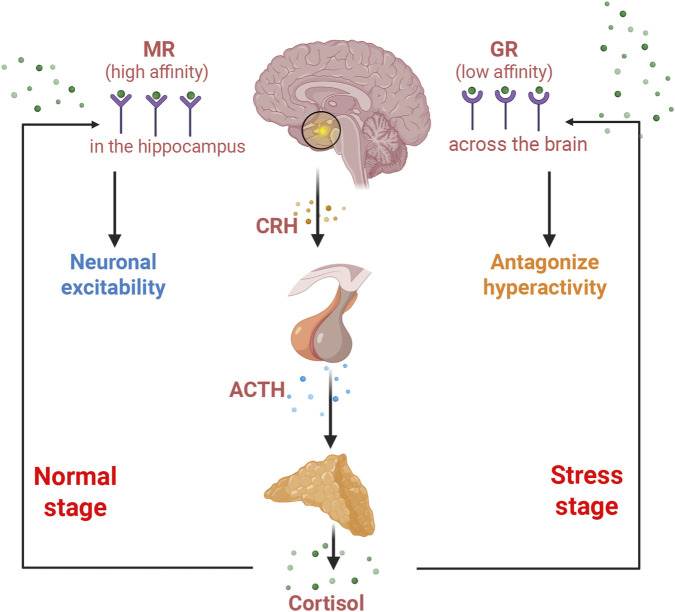
A Diagram illustrates the response of the HPA axis to stress.

The stress response system, particularly the HPA axis, is crucial for mediating responses to physical or psychological challenges. Hyperactivation of the HPA axis is commonly characterized in MDD, marked by elevated levels of cortisol in both plasma and cerebrospinal fluid, exaggerated cortisol responses to adrenocorticotropic hormone (ACTH), hypertrophy of the pituitary and adrenal glands, and impaired negative feedback mechanisms (Figure 3) (Juruena, 2014). As the HPA axis responds to stress, the hypothalamus initially produces corticotropin-releasing hormone (CRH), which travels toward the anterior pituitary gland via the hypophyseal-portal blood to stimulate the secretion of ACTH; releasing into the bloodstream, ACTH subsequently prompts the adrenal gland to secrete glucocorticoids, primarily cortisol in humans and corticosterone in rodents. Cortisol exerts its effects in the brain by binding to two types of receptors: mineralocorticoid receptors (MR) with higher affinity and glucocorticoid receptors (GR) with lower affinity. MR is predominantly expressed in the hippocampus, while GR is more broadly distributed across the brain, including the hippocampus, amygdala, hypothalamus, and brainstem (Keller et al., 2017). The effects of cortisol vary depending on whether it binds to MR or GR, with the outcome influenced by its concentration in the bloodstream. Under normal physiological conditions, cortisol mainly binds to MR in the hippocampus, contributing to functions such as maintaining neuronal excitability. During stress, elevated cortisol leads to increased GR binding, which exerts negative feedback to antagonize HPA axis hyperactivity, thereby limiting further cortisol secretion (Figure 3) (Koning et al., 2019). The balance of MR/GR is vital for cortisol homeostasis and can be significantly impacted by early-life stress experiences (Keller et al., 2017).

The disorder of the HPA axis could be closely associated with dysbiosis of the gut microbiota, dysregulation of mucosal immune responses, and increased intestinal permeability, all of which are characterized by MDD (Zhao and Guan, 2024; Dinan and Cryan, 2012). Firstly, commensal bacteria are implicated in the development and activation of the HPA axis. Germ-free (GF) mice exhibit a more pronounced release of corticosterone and ACTH in response to mild stress compared to specific-pathogen-free controls, which could be reversed by transplantation of commensal bacteria such as *Bifidobacterium infantis* (Sudo et al., 2004; Guo B. et al., 2024). Next, mounting studies have investigated the direct linkage of inflammation and HPA axis activation (Berk et al., 2013; Sudhakaran et al., 2022). Proinflammatory factors like IL-6 are critical in stimulating corticosterone release and HPA activation following immune challenges such as pathogen infections (Zimomra et al., 2011). Intriguingly, cortisol has been found to interact with inflammatory mediators to enhance TDO and IDO expression in the hippocampus, which is pivotal in disturbances of TRP metabolism to engage in the pathogenesis of MDD (Brooks et al., 2016). Additionally, downregulation of MR and GR in the prefrontal cortex and hippocampus has been observed in MDD patients and animal models, which could be either adaptive responses associated with elevated cortisol concentrations or directly mediated by high levels of CRH (Xu et al., 2019; Klok et al., 2011; Medina et al., 2013; Chiba et al., 2012). Notably, inflammatory states may also contribute to the downregulation of these receptors, as evidenced by reduced MR and GR *ex vivo* expression in microglia following LPS-induced inflammation (Sierra et al., 2008). Preclinical and clinical studies have further suggested the roles of MR and GR in the therapeutic effects of antidepressants. It is noted that antidepressants could manage HPA axis hyperactivity by restoring GR functions, while the stimulation of MR as adjuvant therapy can enhance the efficacy of antidepressants (Otte et al., 2010; Anacker et al., 2011). More promisingly, certain effective probiotics have shown the potential to upregulate MR and GR in MDD mice (Wei et al., 2019). Despite these compelling findings, the intricate mechanisms and etiology underlying elevated cortisol levels and MR/GR dysregulation in MDD remain under further investigation.

**FIGURE 4:**
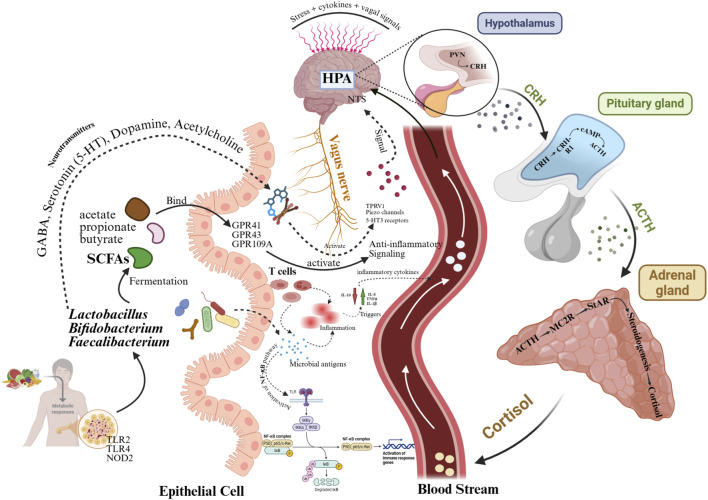
Schematic illustration of the microbiome–immune–neuroendocrine axis, elucidating the interconnected mechanisms by which probiotics modulate the HPA axis and neuroinflammatory pathways via the impact on gut microbiota.

Besides MDD, disorder of the HPA axis is a hallmark feature in various neuropsychiatric conditions, such as anxiety and post-traumatic stress disorder (PTSD). Chronic stress leads to prolonged activation of the hypothalamus (via CRH) and the pituitary (via ACTH), resulting in sustained elevations in cortisol. Over time, elevated cortisol levels impair negative feedback at the glucocorticoid receptor, leading to glucocorticoid resistance and an ongoing state of HPA hyperactivity (Figure 4) (Freimer et al., 2022). This neuroendocrine disturbance is closely intertwined with inflammatory responses: elevated cortisol fails to suppress proinflammatory cytokines (e.g., IL-1β, IL-6, TNF-α), and these cytokines subsequently reactivate the HPA axis, establishing a feed-forward loop that exacerbates these conditions. Moreover, HPA disorder affects gut microbiota composition and intestinal permeability, in which cortisol and stress-related mediators disrupt tight junctions in the gut epithelium, leading to increased translocation of LPS and MAMPs and thereby promoting systemic inflammation and altering the production of microbial metabolites (e.g., SCFAs, kynurenines) (Tan, 2023). The ramifications of these changes extend to the central nervous system, influencing neurotransmitter systems (such as reduced serotonin levels and altered DA/GABA balance), microglial activation, BDNF downregulation, and synaptic plasticity (Shiller, 2025). For instance, it is evident that disruptions in the adolescent-onset HPA axis and gut-brain axis correlate with diminished hippocampal volume, elevated CRH/ACTH ratios, and increased IL-6/IL-10 levels in young adults (Bear et al., 2021). Collectively, investigating the sophisticated network of the HPA axis, immune activation, neurotransmitter disturbances, and gut dysbiosis could offer a comprehensive mechanistic framework. This framework elucidates how probiotic interventions may influence not only gut microbiota but also the broader neuroendocrine-immune-gut axis in relation to mood and stress-related disorders (Prajapati et al., 2025b).

### 2.5 Autonomic nervous system and enteric nervous system in MDD

ANS and ENS serve as essential neural pathways that establish a direct connection between the brain and the gut. The ANS enables bidirectional communication between the ENS and the central nervous system (CNS), transmitting signals from the ENS to the CNS (bottom-up) and conveying information from the CNS to the ENS (top-down). It regulates a variety of digestive functions, including intestinal motility and permeability, bile secretion, luminal osmolarity, and mucosal immune responses, thus maintaining overall physiological homeostasis in the gut (Cryan et al., 2019; Yang Y. et al., 2024). The ANS is made up of two primary divisions: the sympathetic nervous system (SNS) and the parasympathetic nervous system (PNS), which govern the “fight-or-flight” and “rest-and-digest” responses, respectively. In response to stress, the SNS rapidly releases catecholamines like norepinephrine into circulation to prepare the body for damage. Synergistically, norepinephrine can interact with the HPA axis by stimulating CRH release in the hypothalamus, exhibiting inhibitory effects on psychological stress (Dinan and Cryan, 2012). These inhibitory effects provide significant insights into the therapeutic efficacy of SNRIs, which can increase norepinephrine levels to modulate the stress response and alleviate symptoms of MDD, likely associated with dysregulated HPA axis activity. Considered the primary component of PNS, the vagus nerve (VN) acts as a bridge between the brain and the gut through its afferent and efferent fibers that connect to the nucleus tractus solitarii and the enteric nervous system (ENS), respectively (Breit et al., 2018). Interestingly, it has been recently discovered that specialized enteroendocrine cells in the gut could interact directly with the VN, bypassing the ENS and allowing millisecond communication between the gut and the brain (Kaelberer et al., 2018). Beyond its primary role in linking the brain and gut, the VN can also interact with the HPA axis to regulate stress responses and modulate immune functions, particularly by suppressing overall proinflammatory responses (Breit et al., 2018; Bonaz et al., 2017). From this, vagus nerve stimulation (VNS), shown to activate the HPA axis, is being explored as a potential therapeutic approach for conditions with chronic inflammation like MDD (Pavlov and Tracey, 2012; Bermúdez-Humarán et al., 2019).

Mounting evidence has shown that MDD is potentially linked to autonomic imbalance, with a tendency towards increased sympathetic activity and decreased parasympathetic activity (Sgoifo et al., 2015). Autonomic dysfunction, leading to somatic symptoms like cardiac and GI issues, has also been frequently documented among physical complications of MDD patients. Furthermore, the severity of depression is likely to correlate with the extent of autonomic dysfunction (Berger et al., 2011; Kheder et al., 2018). Thus, investigating the mechanisms underlying depression and autonomic dysfunction is imperative for MDD management and treatment, which can provide invaluable insights into the disease process and therapeutic targets. The VN is widely recognized to play a critical role in the pathogenesis of MDD, as research has demonstrated the association between depression and low vagal tone (decreased activity of the VN) (Tan et al., 2022). Clinical trials have also indicated the efficacy of VNS in improving quality of life and reducing scores on depression rating scales like the Hamilton Anxiety Rating Scale (HAMD) and the Self-Rating Depression Scale (SDS). Neuroimaging studies further supported that VNS can positively impact brain activity in regions associated with mood regulation, particularly in cases of treatment-resistant depression (Fang et al., 2016; Conway et al., 2018). Consequently, the U.S. Food and Drug Administration (FDA) has approved VNS as a standard therapeutic approach for treatment-resistant MDD. The mechanisms underlying VNS are multifaceted, including 1. neuronal activation through inducing the expression of neuronal activity markers like c-fos and ΔFosB in specific brain regions involved in regulating the ANS, mood, and stress responses (Cunningham et al., 2008); 2. promotion of anti-inflammatory responses by influencing the immune system (Bottari et al., 2023); 3. elevation of norepinephrine concentration and BDNF expression in the brain (Park et al., 2020); and 4. boosting DA in the NAc alongside 5-HT in the dorsal raphe nucleus (Manta et al., 2013). In addition, Bravo et al. (2011) demonstrated that *Lacticaseibacillus rhamnosus* could lessen depressive-like behaviors in mice, especially in vagotomized models, which further highlights the critical role of the VN in controlling mood disorders via the brain-gut-microbiome axis.

The ENS encircling the GI tract comprises two layers of nerve networks, the submucosal plexus and the myenteric plexus, which collectively control various functions concerning gut motility, digestive juice secretion, blood flow regulation, as well as interaction with the gut immune system (Furness, 2012). It closely resembles the CNS in structure and neurochemistry, allowing it to operate relatively independently and is thus often referred to as the “second brain.” MDD could also be associated with dysfunctional ENS, particularly involved in serotonin signaling (Gershon, 2013). The initial step in serotonin synthesis is catalyzed by the rate-limiting enzyme tryptophan hydroxylase (TPH), which exists in two main isoforms: TPH1 and TPH2. TPH1 is primarily found in peripheral tissues like the intestinal enterochromaffin cells, while TPH2 is specifically expressed in the CNS as well as the ENS (Gershon, 2013; Gershon and Tack, 2007). Although over 90% of serotonin production in the gut relies on TPH1, the 5-HT produced by TPH2 in the ENS is especially crucial for proper ENS development and gut motility (Terry and Margolis, 2017; Israelyan et al., 2019). Notably, gut microbiota can play a critical role in the development and function of the ENS. GF mice display an underdeveloped ENS, which can be restored to maturity through the transplantation of commensal bacteria. This maturation process is intricately linked to serotonin signaling and the activation of the 5-HT4 receptor. Transplanted bacteria promote 5-HT production, which signals the 5-HT4 receptor on enteric neurons, thereby contributing to ENS development and function (Furness, 2012; Bai et al., 2024). Additionally, Toll-like receptor 2 (TLR2), also expressed on enteric neurons, acts as a sensor for bacterial components within the gut. Stimulation of TLR2 by bacteria can trigger the release of Glial Cell Line-Derived Neurotrophic Factor (GDNF) from enteric glial cells, which supports the survival, growth, and differentiation of enteric neurons, aiding in maintaining the structural integrity of the ENS (Brun et al., 2013). Together, understanding how the gut microbiome leverages ENS development could offer valuable insights for developing innovative therapeutic strategies for conditions associated with gut dysbiosis, like MDD.

## 3 Potential probiotics for MDD management via the brain-gut-microbiome axis

The pathophysiological changes characterized by MDD involve a complex interplay among gut microbiota, immune responses, neuroactive substance metabolism, the HPA axis, the ANS, and the ENS. These alterations are interdependent and intricately woven together, reflecting a multifaceted network that contributes to the development and persistence of MDD. This mechanical network underscores the key role of the brain-gut-microbiome axis in depression, highlighting the potential of the gut microbiota as a promising therapeutic target for MDD management (Ye et al., 2024). Numerous animal and clinical studies have demonstrated the significant efficacy of certain probiotics or probiotic-containing foods in managing MDD. A recent meta-analysis involving 1,401 participants found that probiotic supplementation significantly alleviated depressive symptoms and provided a moderate anxiolytic effect. However, there was notable heterogeneity across studies, with variability in treatment duration and probiotic formulations identified as important contributors to the differences in effect sizes (Asad et al., 2025). A substantial population-based cross-sectional study with 26,118 participants reported that individuals in the highest tertile of probiotic exposure had significantly lower odds of experiencing severe depression and a reduced prevalence of depression, especially among men (Kim and Shin, 2019). Beyond established effectiveness in depressive phenotypes, emerging evidence also supports the benefits of probiotics for subclinical populations. In a randomized controlled trial (RCT) involving 39 individuals with subthreshold depression, a 6-week administration of multi-strain probiotics (total daily dose: 4 × 10^9^ CFU) led to decreased plasma serotonin levels, resembling the neurochemical response typically observed with SSRIs (Dacaya et al., 2025). Taken together, current evidence indicates that the effectiveness of probiotics in MDD management could be modulated by multiple factors, including strain specificity, dosing regimn, and treatment duration. The following sections will elaborate on specific probiotic species with documented antidepressant effects and their proposed mechanisms via the brain-gut-microbiome axis (Tables 1, 2). Special emphasis will be placed on *Bifidobacterium* and *Lactobacillus* species, which are among the most extensively studied genera in this context. Additionally, this section will explore next-generation probiotics currently under investigation for their psychobiotic potential and their capacity to restore microbial and immune homeostasis beyond that of conventional strains.

**TABLE 1:** Characteristics of preclinical studies using probiotic interventions targeting depression-related symptoms.

Ref.	Probiotic strain	Model	Dosage and duration	Pathophysiology outcomes
Trudeau et al. (2019)	*B. longum* R0175	Post-MI depression rats	1 × 10^9^ CFU/day, 14 days	Reduce depressive behaviors, CRP, and caspase-3 levels
Savignac et al. (2014)	*B. longum* 1714	Innately anxious mice	1 × 10^9^ CFU/day, 6 weeks	Reduce stress-related behaviors and modulate associated neurobiological pathways
Desbonnet et al. (2010)	*B. infantis* 35624	Juvenile MS rats	1 × 10^10^ CFU/day, 45 days	Improve depressive behaviors and normalize noradrenaline levels
Tian et al. (2019)	*B. infantis* CCFM687	CUMS mice	1 × 10^9^ CFU/day, 5 weeks	Increase 5-HT, 5-HTP, and BDNF levels
Tian et al. (2020)	*B. breve* CCFM1025	CUMS mice	1 × 10^8^ CFU/10 g body weight/day, 6 weeks	Reduce depressive behaviors and increase BDNF expression
Moya-Pérez et al. (2017)	*B. pseudocatenulatum* CECT 7765	Juvenile MS mice	1 × 10^8^ CFU/day, 3 weeks	Reduce HPA axis hyperactivity and systemic inflammation
Agusti et al. (2018)	*B. pseudocatenulatum* CECT 7765	DIO mice	1 × 10^9^ CFU/day, 13 weeks	Reduce TLR2 protein and gene expression, and restore altered 5-HT levels
Duranti et al. (2020)	*B. adolescentis* PRL2019 or *B. adolescentis* HD17T2H	Groningen rats	1 × 10^9^ CFU/day, 5 days	Promote *in vivo* GABA production in the gut to address mood disorders
Guo et al. (2019)	*B. adolescentis*	CRS mice	1 × 10^9^/CFU/day, 3 weeks	Reduce depressive-like behaviors, suppress hippocampal NF-κB signaling, and increase BDNF expression
Xu et al. (2022)	*Lc. rhamnosus* zz-1	CUMS mice	2 × 10^9^/Kg body weight/day, 5 weeks	Restore gut microbiota balance
Xie et al. (2024)	*Lc. rhamnosus* KY16	CUMS mice	1 × 10^10^ CFU/day, 7 weeks	Relieve depressive-like behaviors
Pan et al. (2024)	*Lc. rhamnosus* GG	CEE mice	1 × 10^11^ CFU/kg body weight/day, 3 weeks	Reduce proinflammatory cytokine expression and enhance synaptophysin, PSD-95, and BDNF expression
Ma et al. (2023)	*Lp. plantarum* CR12	CUMS mice	2 × 10^8^ CFU/day, 4 weeks	Reshape the gut microbiota
Zhu et al. (2024)	*Lp. plantarum* JYLP-326	CUMS mice	1 × 10^9^ CFU/day, 3 weeks	Downregulate proinflammatory cytokines and upregulate neuroplasticity-related proteins
Sun et al. (2021)	*Lp. plantarum* WLPL04	CRS mice	1 × 10^10^ CFU/mL in water, 4 weeks	Counteract anxiety- and depressive-like behaviors
Li et al. (2023)	*L. reuteri* 8008	Obesity and depression comorbidity mice	1 × 10^9^ CFU/day, 4 weeks	Ameliorate obesity-related parameters and depressive-like behaviors
Xie et al. (2020)	*L. reuteri* 3	CSDS mice	1 × 10^9^ CFU/day, 4 weeks	Improve gut microbiota composition and benefit serotonin metabolism
Alatan et al. (2024)	*L. helveticus* NS8	WKY rats	1 × 10^9^ CFU/mL in water, 30 days	Alleviate abnormal microbial composition, HPA axis hyperactivity, and depressive-like behaviors
Maehata et al. (2019)	*L. helveticus MCC1848*	sCSDS mice	1 × 10^9^ cells/day, 24 days	Modify sCSDS-induced gene expression patterns in the nucleus accumbens
Wei et al. (2019)	*Lp. paracasei PS23*	Corticosterone-induced depression mice	1 × 10^8^ cells/day, 41 days	Rescue corticosterone-suppressed hippocampal markers and reverse anxiety- and depression-like behaviors
Kim et al. (2020)	*L. mucosae* NK41	*E. coli* K1-induced mice	1 × 10^9^ CFU/day, 5 days	Improve behavioral impairments and correct microbial alterations
Sun et al. (2019)	*L. kefiranofaciens* 2809	CUMS mice	1 × 10^9^ CFU/day, 6 weeks	Enhance exploratory activity and reduce depression-like behaviors while restoring gut dysbiosis
Hao et al. (2019)	*F. prausnitzii* ATCC 27766	CUMS rats	1 × 10^9^ CFU/day, 4 weeks	Increase cecal SCFA concentrations and plasma levels; reduce stress-induced plasma corticosterone, CRP, and IL-6
Ding et al. (2021)	*A. muciniphila* ATCC BAA-835	CRS mice	1 × 10^8^ CFU/day, 3 weeks	Improve depressive-like behaviors, increase β-alanyl-3-methyl-l-histidine and edaravone, and normalize molecular markers
Zhang et al. (2022)	*C. butyricum* RH2	CFSS rats	1 × 10^9^ CFU/day, 17 days	Improve anxiety- and depression-like behaviors and cognitive performance

**TABLE 2:** Characteristics of clinical studies using probiotic interventions targeting depression-related symptoms.

Ref.	Study type	Population	Sample size	Probiotic strain	Dosage and duration	Pathophysiology outcomes
Pinto-Sanchez et al. (2017)	RCT	IBS with diarrhea/mixed stool and comorbid mild-to-moderate anxiety or depression	44	*B. longum* NCC3001	1 × 10^10^ CFU/day, 6 weeks	Reduces depression and increases quality of life
Patterson et al. (2024)	RCT	Impaired sleep	89	*B. longum* 1714	1 × 10^9^ CFU/day, 4 weeks	Reduction in daytime dysfunction, with enhanced social functioning and vitality
Tian et al. (2022)	RCT	MDD	45	*B. breve* CCFM1025	1 × 10^10^ CFU/day, 6 weeks	Improvements in psychometric and gastrointestinal symptoms
Wauters et al. (2022)	RCT	Healthy students	92	*Lc. rhamnosus* CNCM I-3690	2 × 10^11^ CFU/day, 4 weeks	Attenuated stress-induced anxiety and prevented intestinal hyperpermeability to mannitol
Slykerman et al. (2017)	RCT	Pregnancy women	423	*Lc. rhamnosus* HN001	6 × 10^9^ CFU/day, until 6 months postpartum	Reduction in maternal depression and anxiety scores
Ho et al. (2021)	RCT	Insomnia	40	*Lp. plantarum* PS128	6 × 10^10^ CFU/day, 30 days	Decreases in depressive symptoms, fatigue, and cortical excitation, and improvement in sleep quality
Chen et al. (2021)	Open-label trial	MDD	11	*Lp. plantarum* PS128	6 × 10^10^ CFU/day, 8 weeks	Amelioration of depression severity and somatic symptom burden
Rudzki et al. (2019)	RCT	MDD	79	*Lp. plantarum* 299v	2 × 10^10^ CFU/day, 8 weeks	Improved selective attention and verbal learning performance, and increased 3-HKYN:KYN ratio
Godzien et al. (2025)	RCT	MDD	60	*Lp. plantarum* 299v	1 × 10^10^ CFU daily, 8 weeks	Distinct metabolomic responses characterized
Otaka et al. (2021)	Single-arm trial	MDD/bipolar disorder	18	*Lc. paracasei* Shirota YIT 9029	8 × 10^10^ CFU/day, 12 weeks	Reductions in long-chain acylcarnitines and N-acyl taurines, along with increases in oxidized glycerophosphocholine, sphingomyelins, L-histidine, D-valine, and p-cresol
Miyaoka et al. (2018)	Open-label trial	MDD	40	*Clostridium butyricum* MIYAIRI 588	1 × 10^7^ CFU/day, 8 weeks	Achieved a response rate of 70% and a remission rate of 35%

### 3.1 Bifidobacterium longum

Multiple strains of *Bifidobacterium longum* have been studied for the treatment of depression in both animals and humans. Depression is among the critical consequences following myocardial infarction (MI). Utilizing a rat model of MI, a preclinical study indicated that *B. longum* R0175 (administered at 10^9^ CFU daily for 14 days) was effective in reducing plasma C-reactive protein levels, decreasing Caspase-3 activity in the brain, and alleviating depressive-like behaviors subjected to post-myocardial infarction depression (Trudeau et al., 2019). These findings suggested the potential benefits of *B. longum* R0175 in mitigating inflammatory responses and managing post-heart-attack depressive-like symptoms through the brain-gut-microbiome axis. In an RCT trial involving 44 IBS patients with mild to moderate depression, *B. longum* NCC3001 (supplemented at 1 × 10^10^ CFU daily for 6 weeks) was found to significantly improve depressive symptoms and overall quality of life. Notably, patients receiving *B. longum* NCC3001 also exhibited improved neurological responses to negative emotional stimuli in the amygdala and fronto-limbic regions, likely mediated through the impacts of *B. longum* NCC3001 on bacterial metabolism of methylamines and aromatic amino acids (Pinto-Sanchez et al., 2017). Complementing these findings, *B. longum* 1714 showed promising psychobiotic potential across both animal and human studies. In innately anxious BALB/c mice, *B. longum* 1714 (administered at 1 × 10^9^ CFU daily for 6 weeks) could reduce stress-related behaviors and modulate associated neurobiological pathways (Savignac et al., 2014). This strain was further assessed for its influence on stress responses and neural activity during social stress. An RCT involving 89 adults with impaired sleep revealed that supplementation with *B. longum* 1714 (1 × 10^9^ CFU daily) for 4 weeks improved sleep quality and reduced daytime dysfunction due to sleepiness, while enhancing social functioning and vitality after 8 weeks (Patterson et al., 2024). These findings highlight the strain-specific psychobiotic actions of *B. longum* and support its potential as an adjunctive approach for mood and stress-related conditions.

### 3.2 Bifidobacterium infantis

Certain strains of *Bifidobacterium infantis* have been investigated for their potential antidepressant effects in rodent models. With the rat maternal separation (MS) model, a well-established paradigm for studying stress-related GI and mood disorders, chronic administration of *B. infantis* 35624 (1 × 10^10^ CFU in 100 mL of drinking water for 45 days) significantly improved depressive-like behaviors comparable to the SSRI antidepressant citalopram. This preclinical study demonstrated that *B. infantis* 35624 could normalize noradrenaline levels in the amygdaloid cortex and CRH expression in the amygdala, indicating its antidepressant mechanisms via the modulation of the HPA axis and central monoaminergic pathways (Desbonnet et al., 2010). Additionally, *B. infantis* CCFM687 shows promise in a chronic unpredictable mild stress (CUMS) mouse model for alleviating depressive-like behaviors and neuroinflammation. Specifically, this strain (administered at 1 × 10^9^ CFU daily for 6 weeks) was effective in promoting serotonin and BDNF levels in the prefrontal cortex, relieving hyperactivity of the HPA axis, and mitigating plasma corticosterone and proinflammatory cytokines, possibly due to the restoration of gut dysbiosis by enriching beneficial bacteria, such as butyrate-producing genera *Faecalibacterium* and *Roseburia* (Tian et al., 2019). These findings underscore *B. infantis* strains as promising psychobiotic candidates for stress-related mood disorders, functioning through neuroendocrine, immune, and microbiota-mediated mechanisms.

### 3.3 Bifidobacterium breve

The *B. breve* CCFM1025 has also been studied extensively as a promising candidate psychobiotic strain in both animal models and human clinical trials. Within the context of the CUMS mouse model, *B. breve* CCFM1025 exhibited overall antidepressant-like effects comparable to the SSRI antidepressant fluoxetine. Administration of *B. breve* CCFM1025 (1 × 10^9^ CFU daily for 4 weeks) was effective in ameliorating depressive-like behaviors and gut microbiota dysbiosis caused by chronic stress. This strain appears to attenuate inflammatory responses by decreasing serum TNF-α and hippocampal IL-6 levels, as well as mitigating hyperactivity of the HPA axis by reducing corticosterone release and enhancing GR expression. Furthermore, *B. breve* CCFM1025 administration could promote the intestinal production of SCFAs and 5-HT, elevate BDNF expression, and restore c-Fos expression in the brain, further underscoring its potential therapeutic roles (Tian et al., 2020). The psychotropic potential of *B. breve* CCFM1025 was further assessed in an RCT involving 45 MDD patients (Tian et al., 2022). Interestingly, supplementation with *B. breve* CCFM1025 (1 × 10^10^ CFU daily for 6 weeks) resulted in significant improvements in psychometric and gastrointestinal symptoms, evaluated using standard scales such as Hamilton Depression Rating scale-24 Items (HDRS-24), Montgomery-Asberg Depression Rating Scale (MADRS), Brief Psychiatric Rating Scale (BPRS), and Gastrointestinal Symptom Rating Scale (GSRS). Metagenomic and metabolomic analyses revealed that the antidepressant effects of this strain are likely linked to its beneficial impacts on gut microbiome composition and tryptophan metabolism. These studies suggested a practical therapeutic application for *B. breve* CCFM1025 as a complementary treatment for MDD.

### 3.4 Bifidobacterium pseudocatenulatum

The strain *B. pseudocatenulatum* CECT7765 has emerged as a potential therapeutic probiotic for MDD, which warrants further clinical exploration. Utilizing the MS mouse model, administration of *B. pseudocatenulatum* CECT7765 (1 × 10^8^ CFU daily from postnatal days 2–21) could effectively ameliorate hyperactivity of the HPA axis and gut microbiota dysbiosis caused by chronic stress. Notably, this strain was shown to suppress the levels of proinflammatory cytokines like IFN-γ, while reversing the elevated levels of adrenaline in the hypothalamus and catecholamines in the gut (Moya-Pérez et al., 2017). In addition to this study, the antidepressant-like effects of *B. pseudocatenulatum* CECT7765 were also assessed in a diet-induced obesity (DIO) mouse model that recapitulates the metabolic–psychiatric comorbidity, displaying depressive-like behaviors alongside exaggerated HPA axis responses. Interestingly, this specific strain (administered at 1 × 10^9^ CFU daily for 13 weeks) was effective in restoring abnormal levels of adrenaline in the hypothalamus and 5-HT in the hippocampus, as well as elevated TLR2-mediated innate immune activation in both the gut and the hippocampus (Agusti et al., 2018). These preclinical results indicate the potential of *B. pseudocatenulatum* CECT7765 to modulate the neuroendocrine and microbiome-neuronal-immune axis, contributing to the reversal of depressive phenotypes, particularly in the context of obesity or chronic stress.

### 3.5 Bifidobacterium adolescentis

*B. adolescentis* has been suggested to modulate the gut–brain axis through the production of GABA, a key inhibitory neurotransmitter involved in mood regulation. In particular, *B. adolescentis* PRL2019 and *B. adolescentis* HD17T2H, identified as high-GABA-producing strains, were shown to upregulate the expression of GAD genes responsible for GABA synthesis. Administration of *B. adolescentis* PRL2019 (1 × 10^9^ CFU daily for 5 days) or *B. adolescentis* HD17T2H (1 × 10^9^ CFU daily for 5 days) in 5-month-old male wild-type Groningen rats could significantly promote *in vivo* GABA production in the gut, suggesting their potential applications in addressing mood disorders via gut-brain axis interactions (Duranti et al., 2020). Remarkably, *B. adolescentis* could also effectively reduce inflammatory cytokine levels, improve intestinal barrier function, and induce a regulatory mucosal immune response—mechanisms likely contributing to its anxiolytic and antidepressant properties. Using the chronic restraint stress (CRS) mouse model, *B. adolescentis* (administered at 1 × 10^9^ CFU daily for 3 weeks) was found to reduce depressive-like behaviors, lower hippocampal levels of IL-1β, TNF-α, and NF-κB signaling proteins, and increase BDNF expression (Guo et al., 2019). These preclinical studies demonstrated the role of *B. adolescentis* in the gut-brain axis by modulating neurotransmitter production and immune responses, contributing to its potential antidepressant effects.

### 3.6 Lacticaseibacillus rhamnosus

*Lc. rhamnosus* helps foster a beneficial intestinal environment, primarily through the fermentation of monosaccharides like glucose and maltose. Specific strains exhibit significant potential as adjunctive therapies for psychiatric disorders, mediated via multifaceted regulatory mechanisms encompassing gut microbiota composition, intestinal barrier integrity, neuroactive substance metabolism, immune-inflammatory responses, and autonomic functions (Feng et al., 2025).

In the CUMS mouse model, the administration of Lc. rhamnosus zz-1 (2 × 10^9^ CFU/Kg body weight daily for 6 weeks) is effective in alleviating depression-like behaviors, intestinal damage and inflammation, and hyperactivation of the HPA axis. Concurrently, Lc. rhamnosus zz-1 could restore gut microbiota balance by increasing Lachnospiraceae NK4A136 group and Muribaculum while decreasing *Bacteroides*, linking microbial shifts to behavioral and physiological recovery (Xu et al., 2022). Another preclinical study employing the same model demonstrated that Lc. rhamnosus KY16 (administered at 1 × 10^10^ CFU/Kg body weight daily for 7 weeks) could relieve depressive-like behaviors by inducing the production of 5-hydroxytryptophan (5-HTP) from enterochromaffin cells, thereby promoting central synthesis of serotonin (5-HT). Besides, Lc. rhamnosus KY16 is found to upregulate beneficial bacteria (like Akkermansia muciniphila) and the expression of intestinal tight junction proteins including claudin-7, occludin, and zonula occludens-1, while suppressing microglial M1 polarization and systemic inflammation—further contributing to its antidepressant properties (Xie et al., 2024). Notably, the well-investigated strain Lc. rhamnosus GG has been reported to improve cognitive and emotional functioning in a chronic ethanol exposure (CEE) mouse model that induces cognitive deficits and depression-like behaviors. Administration of this strain (1 × 10^11^ CFU/Kg body weight daily for 3 weeks) is noted to reduce the expression of proinflammatory cytokines, including IL-6, IL-1β, and TNF-α, in the ileum, serum, and brain, alongside enhancing the expression of synaptophysin, postsynaptic density protein-95, and BDNF in the hippocampus (Pan et al., 2024).

In addition to these animal studies, clinical trials have further provided compelling evidence supporting the effectiveness of *Lc. rhamnosus* in alleviating symptoms of anxiety and depression across different populations. An RCT enrolled 92 healthy students to assess the potential of *Lc. rhamnosus* CNCM I-3690 for subjective academic stress. It is noted that intake of a dairy product containing *Lc. rhamnosus* CNCM I-3690 (1 × 10^11^ CFU/100 g twice daily for 4 weeks) could significantly attenuate stress-induced anxiety and prevent intestinal hyperpermeability to mannitol (Wauters et al., 2022). Another piece of clinically relevant evidence comes from a larger study targeting perinatal mental health. This RCT trial recruited 423 women at 14–16 weeks of gestation to receive either *Lc. rhamnosus* HN001 (6 × 10^9^ CFU daily) or placebo until 6 months postpartum (Slykerman et al., 2017). Notably, supplementation with *Lc. rhamnosus* HN001 led to a significant reduction in maternal depression and anxiety scores compared to the placebo group, which underscores the potential of this probiotic strain as a preventive or adjunctive intervention for postpartum depression and anxiety, a critical period associated with heightened vulnerability to mood disorders.

### 3.7 Lactiplantibacillus plantarum

Emerging evidence underscores the therapeutic promise of several *Lp. plantarum* strains in mitigating depressive symptoms through modulating the gut-brain microbiome axis. Preclinical studies indicated that *Lp. plantarum* CR12 and *Lp. plantarum* JYLP-326 can significantly alleviate depressive-like behaviors while also restoring intestinal barrier function in mice subjected to CUMS. Specifically, *Lp. plantarum* CR12 (administered at 2 × 10^8^ CFU daily for 4 weeks) demonstrated its capacity to reshape the gut microbiota by diminishing the relative abundance of *Helicobacter pylori*, whereas enhancing levels of *Lactobacillus* species and butyrate-producing microbial populations (Ma et al., 2023); *Lp. plantarum* JYLP-326 (administered at 1 × 10^9^ CFU daily for 3 weeks) was found to downregulate the promoted level of proinflammatory cytokines in both the hippocampus and colon, while significantly upregulating neuroplasticity-related proteins (such as p-TPH2, TPH2, and 5-HT1AR), indicating its role in regulating serotonergic signaling and the balance between inflammation and neuroplasticity (Zhu et al., 2024). Another strain, *Lp. plantarum* WLPL04 (chronically administered at 10^10^ CFU/mL in drinking water for 28 days), was also reported to counteract anxiety- and depressive-like behaviors in the CRS mouse model as well as cognitive deficits induced by chronic stress, mechanistically from the restoration of gut microbiota composition and an increase in key neurobiological markers such as BDNF and TrkB (Sun et al., 2021).

Clinical findings also supported the antidepressant potential of *Lp. plantarum*. For instance, a 30-day oral supplementation with *Lp. plantarum* PS128 (3 × 10^10^ CFU twice daily) could effectively reduce fatigue levels and cortical excitation, further improving deep sleep quality and depressive symptoms in patients suffering from insomnia (Ho et al., 2021). In an 8-week intervention with MDD patients, this strain (3 × 10^10^ CFU twice daily) exhibited marked efficacy in ameliorating both depression severity and somatic symptom burden (Chen et al., 2021). These trial studies demonstrated the good tolerability of *Lp. plantarum* PS128 and its therapeutic promise as a psychobiotic for managing mood disturbances. Another strain, *Lp. plantarum* 299v was accessed as a complementary therapy to SSRI medication in two independent RCTs with adult MDD patients. In one study published in 2019, supplementation with *Lp. plantarum* 299v (1 × 10^10^ CFU twice daily for 8 weeks) could significantly improve selective attention and verbal learning performance, implicating a regulation of the kynurenine metabolic pathway by a reduction in plasma kynurenine levels and an increased 3-HKYN:KYN ratio (Rudzki et al., 2019). The other study published in 2025 further discovered that an 8-week supplementation of *Lp. plantarum* 299v (1 × 10^10^ CFU daily) produced distinct metabolomic responses characterized by greater reductions in long-chain acylcarnitines and N-acyl taurines, alongside increased levels of oxidized glycerophosphocholine, sphingomyelins, L-histidine, D-valine, and p-cresol (Godzien et al., 2025). These outcomes suggested that the psychobiotic effectiveness of *Lp. plantarum* 299v might be mediated through pathways related to mitochondrial function, oxidative stress, inflammation, and microbiota.

### 3.8 Limosilactobacillus reuteri

A substantial body of evidence highlights the effectiveness of *L. reuteri* in modulating the diversity, composition, and metabolism of gut microbiota. Considered as promising treatments, *L. reuteri* alone or contained in multi-strain formulas has been evaluated in addressing depression-like behaviors in animals and depressive symptoms in patients (Cheng et al., 2025). In the CUMS model with obese mice, *L. reuteri* 8008 (administered at 1 × 10^9^ CFU daily for 4 weeks) could significantly ameliorate obesity-related parameters, such as fasting insulin levels and the HOMA-IR index, as well as depressive-like behaviors through rescuing gut dysbiosis. Specifically, this strain was found to restore gut microbial α-diversity and composition, while enhancing the expression of intestinal tight junction proteins, which improves intestinal barrier function and systemic inflammation (Li et al., 2023). Another preclinical study suggested the antidepressant-like effects of *L. reuteri* 3 (administered at 1 × 10^9^ CFU daily for 4 weeks) utilizing the chronic social defeat stress (CSDS) mouse model, a well-established paradigm inducing persistent depressive-like behavior and gut–brain axis dysregulation. Notably, this treatment was shown to improve gut microbiota composition, benefiting serotonin metabolism likely via a gut-derived serotonergic pathway. In detail, this strain could counteract the stress-suppressed 5-HT levels in both the blood and colon, mechanistically involved in the upregulation of enzymes (like TPH1) for serotonin biosynthesis while inhibiting those (like IDO) for its catabolism (Xie et al., 2020).

### 3.9 Lactobacillus helveticus

*L. helveticus* was shown to ameliorate the imbalanced composition of gut microbiota and dysfunction of the brain-gut-microbiome axis, which are considered key contributors to endogenous depression. Wistar-Kyoto (WKY) rats are employed as an endogenous model for studying depression, particularly treatment-resistant depression (TRD), characterized by a disrupted brain-gut-microbiome axis. In this context, the chronic administration of *L. helveticus* NS8 (1 × 10^9^ CFU/mL in drinking water for 30 days) was found to alleviate the abnormal microbial composition, hyperactivity of the HPA axis, and depressive-like behaviors. Neurobiologically, this strain appears to increase levels of GR, BDNF, DA, and 5-hydroxyindoleacetic acid (5-HIAA) in the hippocampus, while inhibiting CRH in the hippocampus and norepinephrine in the hypothalamus, all of which are correlated with the depressive-like behaviors as well as serotonergic and noradrenergic neurotransmission (Alatan et al., 2024). Moreover, pasteurized *L. helveticus* MCC1848 (administered at 1 × 10^9^ cells daily for 24 days) also demonstrated antidepressant effects, which have been examined in the subchronic and mild social defeat stress (sCSDS) mouse model. Interestingly, this intervention could modify the sCSDS-induced gene expression patterns in the NAc, a critical brain region for stress-resilience, potentially contributing to the balance of dopaminergic and serotonergic neural functions (Maehata et al., 2019).

### 3.10 Lacticaseibacillus paracasei

Repeated injections of corticosterone, the principal stress hormone in rodents, can disrupt the HPA axis, leading to depression and anxiety phenotypes in mice. Using this model, both live and pasteurized *Lc. paracasei* PS23 (administered at 1 × 10^8^ cells daily for 41 days) were examined, and the results indicated that both forms could rescue the corticosterone-suppressed hippocampal levels of BDNF, MR, and GR, further reversing anxiety- and depression-like behaviors. Interestingly, live *Lc. paracasei* PS23 was found to restore 5-HT levels in the hippocampus, prefrontal cortex, and striatum, whereas the pasteurized form specifically improved dopamine levels in the hippocampus and prefrontal cortex (Wei et al., 2019). Further, a single-arm clinical trial involving 18 patients with MDD or bipolar disorder (BD) demonstrated that a 12-week supplementation of *Lc. paracasei* Shirota YIT 9029 (8 × 10^10^ CFU daily) exhibited pronounced therapeutic effects on depressive symptoms associated with the modulation of gut microbiota composition, primarily through the *Actinobacteria* phylum (Otaka et al., 2021).

### 3.11 Limosilactobacillus mucosae

Oral gavage of *Escherichia coli* K1, a strain known for high LPS production, has been determined to induce gut dysbiosis, accompanied by cognitive decline and depressive-like behaviors in mice. In this context, the administration of *L. mucosae* NK41 (1 × 10^9^ CFU daily for 5 days) demonstrated a significant improvement in both behavioral impairments and microbial alterations. Specifically, *L. mucosae* NK41 could lead to an increased abundance of beneficial *Lactobacillaceae*, *Eubacteriaceae*, and *Bacteroidaceae*, while preserving intestinal barrier integrity by upregulating tight junction proteins claudin-1 and occludin. Additionally, this intervention was shown to suppress K1-induced LPS and inflammation levels in the gut and systemic circulation, further exhibiting neuroprotective effects by promoting the expression of BDNF and the phosphorylation of cAMP response element-binding protein in the hippocampus (Kim et al., 2020).

### 3.12 Lentilactobacillus kefiranofaciens

A preclinical study using the CUMS mouse model investigated the antidepressant effects of *L. kefiranofaciens* CGMCC2809, a novel probiotic strain isolated from Tibetan kefir grains. The findings indicated that *L. kefiranofaciens* CGMCC2809 (administered at 1 × 10^9^ CFU daily for 6 weeks) could significantly improve exploratory activity and depression-like behaviors, alongside restoring gut dysbiosis with an increased level of anti-inflammatory bacteria and a reduction of stress-related microbial populations like *Proteobacteria*. Remarkably, this strain demonstrated effectiveness in relieving the dysregulated HPA axis and elevating serum tryptophan and hippocampal 5-HT levels (Sun et al., 2019).

### 3.13 Next-generation probiotics

While predominant studies on probiotics for MDD have concentrated on featuring species of *Bifidobacterium* and *Lactobacillus*, recent advancements have expanded this scope to include “next-generation probiotics” (NGPs). Among these, *Faecalibacterium prausnitzii, Akkermansia muciniphila,* and *Clostridium butyricum* have shown antidepressant and anxiolytic effects in both animal models and human studies. These species are commensals with specialized immunoregulatory and neuroactive functions, capable of restoring gut-brain-immune homeostasis through various processes such as SCFA production, mucin degradation, and the modulation of microglial and vagal signaling.

One dominant gut butyrate-producing species, *Faecalibacterium prausnitzii,* exhibits potent anti-inflammatory and neuroprotective properties, suggesting its critical role in the intricate microbiome-neuronal-immune axis through the modulation of inflammation and neurotransmitter synthesis (Liu et al., 2023a). Notably, the depletion of this bacterium is linked to the severity of MDD, with preclinical evidence showing the potential of specific strains in depressant-like behaviors. For instance, *F. prausnitzii* ATCC 27766 (administered at 1 × 10^9^ CFU daily for 4 weeks) exhibited both preventive and therapeutic effects in the CUMS rat model. This strain was found to promote cecal SCFA concentrations and plasma levels of IL-10, as well as reducing stress-induced plasma corticosterone, C-reactive protein, and IL-6 (Hao et al., 2019).

Another key species in the gut microbiome consistently associated with MDD is *Akkermansia muciniphila*, which can generate multiple SCFAs (such as acetate, propionate, butyrate, isobutyric acid, and isovaleric acid) as it ferments mucin in the host intestinal mucus layer (Li et al., 2021; Lei et al., 2023). In a mouse model of CRS, administration of *A. muciniphila* ATCC BAA-835 (1 × 10^8^ CFU daily for 3 weeks) could significantly improve depressive-like behavior, associated with increased levels of β-alanyl-3-methyl-l-histidine and edaravone and restored abnormal variations in molecular markers related to depression, including corticosterone, dopamine, and BDNF (Ding et al., 2021). The antidepressant effects of this strain have also been studied in the CUMS mouse model. Interestingly, this intervention significantly increased the level of serotonin (5-HT) and inhibited the expression of the serotonin transporter (SERT) in the gut, thereby modulating gut-to-brain signaling through suppression of enteric nerve activation (Guo H. et al., 2024).

It is reported that both humans and animals could experience a reduction in *Clostridium butyricum* as affected by depression (Liu et al., 2024). This species is also known to produce SCFAs, particularly butyrate, which is vital for maintaining gut barrier integrity and reducing neuroinflammation. Administration of *C. butyricum* RH2 (1 × 10^9^ CFU daily for 17 days) demonstrated significant improvements in anxiety- and depression-like behaviors, as well as cognitive function in rats subjected to chronic foot shock stress (CFSS). This intervention could lead to decreased serum ACTH and corticosterone, along with increased BDNF levels in the hippocampus (Zhang et al., 2022). In clinical settings, supplementing *C. butyricum* MIYAIRI 588 (20 mg of 1 × 10^7^ CFU/g twice daily for the first week, then three times daily from week 2 to 8) achieved a response rate of 70% and a remission rate of 35% among patients suffering from treatment-resistant MDD, underscoring its potential as an adjunctive therapy. These results emphasize the promising role of NGPs in managing MDD and the necessity for continued research in both preclinical and clinical cohorts (Miyaoka et al., 2018).

## 4 Complementary approaches to probiotic interventions

Beyond probiotics, a range of microbiome-based therapies have also been explored for their effects on depression and related mood disorders, including prebiotics, synbiotics, postbiotics, and fecal microbiota transplantation (FMT). These therapies act along a continuum of complexity and potency in modulating the gut microbiome, from providing nourishment for beneficial bacteria to introducing an entire living microbial ecosystem. As complementary approaches to probiotics, these interventions target distinct yet overlapping microbiome-neuronal-immune pathways (Smolinska et al., 2025; Ghosh and Sands, 2025).

Prebiotics are non-digestible carbohydrates, such as specific oligosaccharides or fibers, which are indigestible for the human body but can stimulate beneficial taxa to modify microbial composition in the gut (Khaledi et al., 2024). Specifically, these compounds can influence the growth and metabolism of probiotics, which enhances the production of SCFAs and other critical metabolites relevant to gut-brain communication, closely associated with the pathophysiology of MDD (Liu et al., 2023b; Dai et al., 2025; Dziedzic et al., 2024). Using a chronic unpredictable social stress (CUS) mouse model, the combined administration of fructooligosaccharides and galactooligosaccharides (FOS + GOS dissolved in drinking water for 0.3–0.4 g/mouse daily) over 10 weeks could result in the alleviation of depressive and anxiety-like behaviors, accompanied by the normalization of stress-related endocrine, immune, and microbial alterations (Burokas et al., 2017). These preclinical findings suggest that prebiotics could have psychotropic effects, likely mediated via increased SCFA production and tryptophan availability; however, the extent of their clinical antidepressant effects remains uncertain. A meta-analysis including 34 RCTs reported no significant improvement in depressive or anxiety symptoms attributable to prebiotics, whereas probiotics demonstrated evident therapeutic benefits (Liu et al., 2019). Similarly, a more recent meta-analysis of 13 RCTs also concluded that prebiotics alone did not yield significant improvements in depressive symptoms as well (Zhang et al., 2023). Recent reviews noted that the biological effects induced by prebiotics are generally modest and variable across studies, further suggesting that their impact on depression may be limited under current intervention conditions (Zhu et al., 2025; Wang M. et al., 2024).

Synbiotics combine prebiotics and probiotics in a formulation designed to function synergistically, with prebiotics serving as the substrates or nutrients that enhance the survival, colonization, growth, and metabolic capabilities of probiotics in the gut (Al-Habsi et al., 2024). This coordinated action could be more effective than using prebiotics or probiotics alone in reshaping the gut microbiota, increasing beneficial metabolites (e.g., SCFAs), and improving the intestinal barrier and immune homeostasis (Dai et al., 2025; Ciernikova et al., 2023). An RCT investigated the effectiveness of synbiotics as an adjuvant therapy to the SSRI antidepressant fluoxetine in 40 adults with moderate depression. This study reported that a 6-week supplementation of synbiotic capsules (containing seven probiotic strains at a total of 4.3 × 10^9^ CFU plus 200 mg FOS daily) exhibited a greater reduction in depression scale scores, complementary to the antidepressant effectiveness of fluoxetine (Ghorbani et al., 2018). Further, the most recent meta-analysis of 19 RCTs involving 1,405 participants demonstrated that synbiotics could generate significant improvements in standardized measures of depression and anxiety symptoms (Moshfeghinia et al., 2025). Despite these encouraging findings, the therapeutic efficacy of synbiotics remains highly formulation-dependent, as substantial heterogeneity in probiotic strains, prebiotic components, and dosing regimens continues to limit the generalizability of the current evidence.

Postbiotics refer to non-viable microbial cells, cellular components, or metabolic byproducts that confer health benefits to the host by modulation of the gut–brain axis. These substances include inactivated microorganisms, cell wall fragments (e.g., peptidoglycan and lipoteichoic acid), secreted metabolites (like SCFAs), bioactive peptides, vitamins, and polysaccharides (Chudzik et al., 2021). Emerging evidence suggests that postbiotics could ameliorate depressive symptoms and support neuroprotection through multiple interconnected biological pathways, including oxidative stress, mitochondrial function, neurotransmitter synthesis, neuroinflammation, and neuroplasticity, all of which are implicated in the pathophysiology of depression (Sarkar et al., 2025). In a preclinical study employing postbiotics derived from *Lp. plantarum* HJZW08, a 15-day pretreatment with heat-killed bacteria or bacterial metabolites (2 × 10^8^ CFU or metabolite-equivalent supernatant daily) could effectively prevent neurological dysfunction induced by pathogen challenges. This treatment likely influences the intricate microbiome-neuronal-immune axis by promoting beneficial taxa (like *Lactobacillus* and *Dubosiella*) and reducing harmful taxa (like *Mucispirillum*), preserving SCFA levels in the gut, regulating the metabolism of neuroactive substances (such as serotonin, dopamine, GABA, and BDNF), alleviating neuroinflammation (evidenced by decreased proinflammatory factors and increased anti-inflammatory factors), and thereby leading to the improvements in depression- and anxiety-like behaviors as well as cognitive performance (Wu Y. et al., 2022). Research on postbiotics in the context of depression is still in the nascent stages, with very scarce RCT findings available to support robust meta-analytic evaluation (Dai et al., 2025). Future research should aim to clarify the bioactive components driving psychobiotic effects, establish optimal intervention strategies, and incorporate postbiotics into larger multi-arm clinical trials alongside probiotics, prebiotics, and synbiotics for comparative efficacy evaluation.

FMT offers a more radical ecosystem-level intervention where fecal microbiota from a healthy donor is transferred to the gastrointestinal tract of a patient to reconstruct the beneficial composition, diversity, and functions of gut microbiota. To this end, this medical procedure has the potential to improve intestinal barrier integrity, mitigate systemic inflammation, modulate neuroendocrine systems, and ultimately restore the overall gut-brain axis signalling. These mechanisms collectively indicate a promising therapeutic role for FMT in addressing depression and other neuropsychiatric disorders. Interestingly, an RCT, involving a cohort of 40 COVID-19 patients with gastrointestinal symptoms and depressive features, demonstrated that intake of 10 flora capsules daily for four consecutive days (in total about 200 g of fecal material) could significantly alleviate depression (*p* = 0.006) in 1 week (Jiang et al., 2024). The antidepressant promise of FMT was further supported by the most recent meta-analysis of 12 RCTs investigating FMT interventions for depressive symptoms (Zhang et al., 2025). However, the long-term safety profile of FMT is still inadequately defined, and the current clinical evidence supporting the use of FMT for depression remains limited. In particular, standardization presents a significant challenge, as there is considerable variability in donor selection, preparation protocol, delivery method, and microbiota characterization. To ascertain the therapeutic value of FMT in the context of MDD, future research should prioritize large-scale, well-controlled trials with rigorous donor screening, validated endpoints, and a thorough exploration of underlying mechanisms.

## 5 Limitations and critical points

Current research on probiotics for MDD presents several notable limitations. Many preclinical studies use small sample sizes, limiting statistical power. The heterogeneity in probiotic formulations, dosages, and study durations complicates cross-study comparisons. In addition, inconsistent outcomes observed in different animal models, potentially influenced by variations in stress paradigms, host interactions, and disease pathophysiology, make it more challenging for mechanistic interpretations. In clinical settings, probiotic supplementation for MDD patients often lacks long-term follow-up and large cohorts. Also, the variability in gut microbiota profiles across different populations could significantly hinder the generalizability of efficacy assessments for probiotics. Further, some trials utilized multi-strain probiotic formulations, making it difficult to determine the strain-specific contributions due to the complex interactions involved. Critical points should include the need for methodologically rigorous approaches, mechanistic clarity regarding strain-specific effects, and robust clinical trials to integrate mechanistic endpoints from preclinical findings, as well as assess long-term safety and efficacy.

## 6 Conclusion and perspectives

The brain-gut-microbiome axis plays a central role in the onset and progression of MDD, mechanistically through a multifaceted interplay of immune, metabolic, endocrine, and neural pathways. Both preclinical and clinical investigations have demonstrated that specific probiotic strains—particularly from the genera *Bifidobacterium* and *Lactobacillus*—could alleviate depressive symptoms by restoring microbiota composition, suppressing proinflammatory cytokines, enhancing the metabolism of neuroactive substances like serotonin, dopamine, and GABA, attenuating hyperactivity of the HPA axis, and promoting neuroplasticity. Remarkably, certain strains have even shown efficacy comparable to conventional antidepressants, underscoring the therapeutic promise of probiotic supplements in the management of MDD. Despite these compelling discoveries, current clinical evidence remains limited due to small sample sizes, heterogeneity in probiotic formulations, and variability in study designs. Future research should prioritize large-scale, multi-center, prospective trials, which ought to incorporate antidepressant treatment as positive control groups in order to compare directly the efficacy of probiotics and to explore in-depth the advancements of combinatorial regimens where probiotics are administered in conjunction with antidepressants.
